# Occupational hazards: Perceptions of nurse managers at Intermediate Hospital Onandjokwe

**DOI:** 10.4102/hsag.v30i0.2904

**Published:** 2025-06-10

**Authors:** Kristofina T. Nakatana, Hans Justus Amukugo, Salomo Salomo

**Affiliations:** 1 Department of General Nursing, Faculty of Health Sciences and Veterinary Medicine, University of Namibia, Windhoek, Namibia

**Keywords:** healthcare facility, management, nurses, occupational health hazards, perceptions

## Abstract

**Background:**

Managing occupational health hazards (OHH) in the nursing profession is crucial for improving nurses’ quality of life. Nurses are facing OHH while delivering patient care. These hazards can lead to morbidity, mortality and compromised patient care.

**Aim:**

The study explored and described the perceptions of nurse managers regarding the management of OHH among nurses at the Intermediate Hospital Onandjokwe.

**Setting:**

The study was conducted in the state healthcare facility at the Intermediate Hospital Onandjokwe.

**Methods:**

A qualitative approach with phenomenological, descriptive, exploratory and contextual designs was adopted. Non-probability purposive sampling was employed, and sample size was determined by data saturation. The study utilised an interviewer guide, field notes and an audio recorder as instruments for data collection. The data were analysed using a thematic approach. The criteria for ensuring data trustworthiness were applied, and fundamental ethical principles were followed.

**Results:**

Three major themes and their corresponding sub-themes emerged from the data, such as: (1) perceived factors associated with the management of OHH, (2) experiences of OHH and (3) challenges faced by nurses in managing OHH.

**Conclusion:**

The nurse managers perceived poor management of OHH in the hospital, which exposed nurses to physical, chemical, biological and psychosocial health hazards.

**Contribution:**

The study supports the sustainable development goals by improving health and safety practices for nurses and enhancing their working conditions. It aligns with Vision 2023 by promoting a healthier, safer workforce to improve nurses’ health and well-being.

## Introduction

Occupational health hazard (OHH) is defined as any workplace condition that causes a risk to the employee’s health during or after being employed in any industry. They are classified into biological and non-biological health hazards (Selvi 2020). Nurses are exposed to injuries and disease while discharging their duties, which can result in mortality and morbidity (Gbadago, Amedome & Honyenuga 2017). The OHH has a negative impact on nurses’ well-being and can compromise the quality of patient care (Che Huei et al. 2020). The World Health Organization (WHO 2021) emphasised that nurses need to work in a safe environment to achieve the Sustainable Development Goal 8 (SDG-8). The OHH can only be managed if there is a higher level of knowledge of the causes and management measures at the workplace, such as administrative policy and the adequate and effective utilisation of personal protective equipment (PPE) (Ndejjo et al. 2015).

Globally, 374 million people are suffering from occupational diseases and injuries annually, with 2.78 million deaths (International Labour Organization [ILO] 2022). Subsequently, the WHO (2020) revealed that 136 million nurses work in healthcare facilities globally. Furthermore, nurses are at risk of OHH and 1 million have succumbed to OHH annually (Galam 2018). In Iran, 89% of the nurses have reported musculoskeletal injuries (Sabita et al. 2018). The causes of the prevalence of OHH are a lack of knowledge among nurses, poor management and negligence of the employers (Chowdhury & Chakraborty 2017). The common biological and non-biological dangers experienced by nurses’ managers were verbal abuse, cuts, wounds, infections from patients, chemical inhalation, lower back discomfort and injuries connected to sharp objects (Alhassan & Poku 2018).

In Africa, nurses experienced OHH in health facilities, namely clinics, health centres and hospitals. Around 93% of the nurses in Ghana have experienced OHH, such as physical health hazards, followed by 20% biological and 17% psychosocial (Alhassan & Poku 2018). In Uganda, 50% of the nurses experienced OHH because of a lack of the necessary PPE, working overtime and job-related pressures (Ndejjo et al. 2015). In South Africa, more than 50% of the nurses report being infected with latent tuberculosis (TB), which puts them at risk of developing multidrug resistance (Naithani et al. 2021). The contributing factors to these hazards are a need for more awareness of occupational health and safety policy among nurses, ignorance and underfunding by the government, ineffective technical skills and political commitment (Aluko et al. 2016). Namibia has reported 28% of the cases of occupational injuries between 2010 and 2018, and the Ministry of Health and Social Services (MOHSS) reported 4% of the cases of OHH between 2010 and 2018 (Ministry of Labour, Industrial Relations and Employment Creation [MLIREC] 2021).

### Problem’s statement

The study was prompted by a rise in sick leave at Intermediate Hospital Onandjokwe between 2020 and 2022, with 3702 cases reported among nurses, attributed to stress and burnout from OHH (MOHSS 2022). Occupational health hazards among nurses are regarded as a major public health concern, and nurses are facing an enormous challenge to manage hazards (Ndejjo et al. 2015). Rai et al. (2021) stated that there is a global increase in the number of cases of OHH among nurses in health settings. Occupational health hazard is under-reported because of inadequate research, lack of resources and lack of capacity development (Ogunnaike & Akinwaare 2020). Regardless of the existence of the National Occupational Safety and Health (OSH) policy, challenges such as insufficient PPE and poor coordination among OSH activities are still being observed in health facilities (MLIREC 2021). Despite the policy’s existence, much is not known about the extent to which nurses at Intermediate Hospital Onandjokwe are complying with written protocols designed to prevent and mitigate health hazards.

### Aim of the study

The study aimed to explore and describe the perceptions of nurse managers regarding the management of OHH among nurses in the state healthcare facility in the Intermediate Hospital Onandjokwe.

### Research setting

The study was conducted at the Intermediate Hospital Onandjokwe, located in the Oshikoto region of Namibia. Namibia, situated in southwestern Africa, spans approximately 824 000 km^2^ (Demographic Dividend Study Report 2018). The Intermediate Hospital Onandjokwe is a semi-rural healthcare facility in Oniipa town, in the northwestern part of the country. It was established in 1908 by Lutheran Finnish missionaries under the leadership of Dr. Selma Rainio; it holds the distinction of being the first hospital in northern Namibia. It is also the third-largest hospital in the country, following those in Windhoek and Oshakati.

## Research methods and design

The study adopted a qualitative research design with phenomenological, exploratory, descriptive and contextual designs. These designs were used to explore and describe the perceptions of nurse managers regarding the management of OHH among nurses in the state healthcare facility in the Intermediate Hospital Onandjokwe. The study employed a qualitative research approach, integrating phenomenological, exploratory, descriptive and contextual designs. The study aimed to explore and describe nurse managers’ perceptions of managing OHH among nurses at the Intermediate Hospital Onandjokwe. The qualitative approach was chosen for its ability to capture and convey the lived experiences of participants by providing a deeper understanding of the phenomenon under investigation. The qualitative approach was chosen to promote effective strategies for managing OHH in nursing practice and to gain insights into the challenges and practices within this context. The phenomenological design was used to explore the subjective perceptions and lived realities of nurse managers regarding OHH management (Balikçi 2019). An exploratory design was also employed to enhance understanding of the phenomenon, particularly because of the limited existing literature on OHH management (Hunter, McCallum & Howes 2019). Moreover, the contextual design was used to facilitate the collection of nuanced data, allowing for a more empathetic and comprehensive understanding of participants’ experiences (Creswell & Creswell 2017).

### Paradigm perspective

The study used a paradigm as its philosophical framework to guide the research. This paradigm helped in understanding and interpreting the social phenomena being investigated (Creswell & Creswell 2017). The following meta-theoretical assumptions, such as ontological, epistemological, axiological, methodological and rhetorical, were used in the study (Creswell & Creswell 2017). The study adopted interpretivism, assuming that reality is subjective, multiple and socially constructed (Alharahsheh & Pius 2020).

### Population and sampling

The nursing population at Intermediate Hospital Onandjokwe comprised 12 nurse managers, selected based on specific inclusion criteria. These included the Control Registered Nurse, Chief Registered Nurse of Maternity, Chief Registered Nurse of the Emergency Unit, Chief Registered Nurse of the Patient Department, Chief Registered Nurse of Theatre, Chief Registered Nurse of Outpatient Departments, Administrator Registered Nurse, Information and Health Systems Senior Registered Nurse, Senior Registered Nurse in the Medical Ward, Senior Registered Nurse in the Surgical Ward, Senior Registered Nurse in the Maternity Ward and Senior Registered Nurse in the Casualty. Purposive sampling was used because of the small population of nurse managers and their expertise in the studied phenomenon (Polit & Beck 2021). As a result, eight nurse managers were individually interviewed, and data saturation was reached.

The following inclusion criteria were applied during sampling:

- Participants must be registered nurses holding managerial positions at the Intermediate Hospital Onandjokwe.
- Participants must be in a managerial position for more than 3 years.
- Participants must be on a voluntary basis.

### Data collection

Data were collected using semi-structured face-to-face interviews with an interview guide, field notes and audio recorders. The researcher planned with each manager before data collection and agreed on the date and time of the interviews. A consent letter was given to participants to sign before the interviews. Before the interview commenced, the aim and benefits of the study were explained to the participants. Participants signed the consent for voluntary participation in the interviews. The researcher explained all the ethical issues, such as confidentiality, anonymity, the use of an audio recorder, privacy, beneficence and non-maleficence, before interviews (Brink, Van der Walt & Van Rensburg 2018). All interviews were conducted in each manager’s private office.

The researcher opted for in-depth face-to-face interviews by using an interview guide, and a central question was posted to the participant as follows: The main question of the study was: *Can you please tell* me *how you perceive the management of occupational health hazards among nurses in this health facility*? Probing questions were used depending on the response of the respondent to get more clarity on the response provided. During the interview, the field notes were taken by the researcher to capture non-verbal and verbal communications with the purpose of data enhancement and providing a rich context for data analysis. The non-verbal aspects taken from participants were such as eye contact, gesture, posture and voice tone (Phillippi & Lauderdale 2018). Techniques for both verbal and non-verbal communications were applied during the study to encourage nurse managers to articulate their perceptions about the management of OHH among nurses at the Intermediate Hospital Onandjokwe. Probed questions were given to participants for complexity and accuracy when additional information about their previous responses did not entail inadequate information for the question given (Robinson 2023).

### Data analysis

Data analyses were done by thematic approach analysis, and six steps of the coding process were followed (Naeem et al. 2023). The steps were carried out as follows:

- *Step 1: Transcription and familiarisation* – This step involved transcribing the data and becoming familiar with it. It also included selecting quotations that represented the lived experiences of nurse managers, helping to understand their perspectives through their words. The themes were then obtained inductively from the data.
- *Step 2: Keyword selection* – In this step, keywords were selected from the interviews to highlight key concepts and ideas that would help in further analysis.
- *Step 3: Coding* – The coding step involved assigning specific codes to segments of the data. Each code represented a particular theme or concept that emerged from the data.
- *Step 4: Theme development* – This step involved organising the codes into groups by identifying patterns and relationships between them. This helped to develop broader themes from the data.
- *Step 5: Conceptualisation* – The conceptualisation step focuses on interpreting the keywords, codes and themes to derive meaningful insights and understanding from the data.
- *Step 6: Conceptual model development* – In this final step, a conceptual model was developed based on the interpreted data. The final themes and subthemes were formulated to provide a clear representation of the findings.

### Measures to ensure the trustworthiness of the data

To enhance the trustworthiness of qualitative data, the study has adopted the following criteria, namely: credibility, dependability, confirmability, transferability and authenticity (Havenga 2019). The credibility was used to ensure accuracy and truthfulness to the participants’ experiences. The credibility was achieved through prolonged engagement with participants and creating rapport before the interviews commenced. Additionally, the data were validated through debriefing, discussions and replaying of the audio with participants to confirm the accuracy. Dependability was used to ensure consistency and stability of the data over time in the research process. This was enhanced by piloting an interview guide to ascertain whether participants understood the questions and whether the questions elicited appropriate discussions. The pilot study was conducted at the Intermediate Hospital Oshakati and involved interviews with four nurse managers. The pilot study results were excluded from the main study analysis. The interviews were audio recorded and subsequently audited to assess their relevance and applicability. To ensure confirmability, rigorous review of interview transcripts was done to guarantee that research findings were shaped by the participants and not influenced by the researcher’s bias. Transferability was ensured by linking the study findings with description, methodology and sampling to similar findings of research studies conducted globally. Concerning authenticity, interviews were audio recorded to capture participants’ experiences. Similarly, the report of the study contains direct excerpts from the participants portraying the lived perception regarding the management of OHH to indicate the true representation of diverse perspectives (Havenga 2019).

### Ethical considerations

Approval to conduct the study was obtained from the University of Namibia Decentralised Ethical Committee DEC (reference no: OSH-0081) and the ethical clearance certificate (reference no: SoNPHHDB/23/116/117). Further approval to conduct the study (reference no: 22/4/2/3) was provided by the MOHSS. Participants provided written informed consent after understanding the study’s purpose. The research adhered to ethical principles from the Declaration of Helsinki, and following research fundamental ethical principles of the World Medical Association (WMA) guided the study, as stipulated in the including respect for persons, beneficence and justice (Parsa-Parsi 2022). Data were anonymised through coding to ensure participants’ identities were protected, and no one was forced to participate.

## Results

The study included eight nurse managers from the Intermediate Hospital Onandjokwe, with Table 1 presenting the demographic characteristics of the participants.

**TABLE 1:** Demographic characteristics of the participants.

Variables	Characteristics	Frequencies	Percentages (%)
Age (years)	46–50	3	37.5
	51–55	4	50.0
	56–60	1	12.5
Gender	Male	1	12.5
	Female	7	87.5
Positions	Chief Registered Nurse	4	50.0
	Senior Registered Nurse	4	50.0
Units of work	Theatre	1	12.5
	Inpatient officer	1	12.5
	Matron officer	2	25.0
	Training officer	1	12.5
	Medical ward	1	12.5
	Surgical ward	1	12.5
	Maternity ward	1	12.5
Years of experience	From 10 to 20 years	1	12.5
	From 21 to 30 years	5	62.5
	From 31 to 40 years	2	25.0

Data analysis revealed three primary themes: (1) perceived factors associated with the management of OHH, (2) experiences of OHH in health facilities and (3) challenges faced by nurses in managing OHH in health facilities. Table 2 provides the foundation for discussing the themes and sub-themes identified through data analysis. To protect participants’ identities, data were anonymised using coding, and participation was voluntary.

**TABLE 2:** Perception of the nurse managers regarding the management of occupational health hazards in the health care facility.

Themes	Sub-themes
1. Perceived factors associated with the management of occupational health hazards	1.1 Inadequate management of occupational health hazards 1.2 Inadequate training and education related to the management of occupational health hazards 1.3 Lack of awareness of the management of occupational health hazards 1.4 Absence of policies and guidelines related to the management of occupational health hazards
2. Experiences occupational health hazards in health facilities	2.1 Biological hazards 2.2 Psychological hazards 2.3 Physical hazards
3. Challenges faced by nurses in managing occupational health hazards in health facilities	3.1 High prevalence of occupational health hazards 3.2 Shortage of human and material resources 3.3 Non-adherence to safety measures among nurses 3.4 Overcrowding of student nurse

### Theme 1: Perceived factors associated with the management of occupational health hazards

The participant’s perceptions were explored regarding the management of OHH. Subsequently, participants revealed the following perception of managing OHH in healthcare facilities and were divided into subthemes, namely inadequate management of OHH, inadequate training and education related to the management of OHH, lack of awareness about the management of OHH and absence of policies and guidelines related to the management of OHH. As a result, the sub-theme is being discussed hereunder.

#### Sub-theme 1.1: Inadequate management of occupational health hazards

The study examines the perceived factors related to the management of OHH, with a specific focus on the inadequate management of these hazards that obstruct effective interventions within healthcare settings. The participants provided the following insights:

‘An occupational health hazard management is a neglected area that needs to be taken care of in this hospital and it is a major public concern.’ (P1, female, 49 years old)

‘My perception of the management of occupational health hazards is very poor in this hospital.’ (P6, female, 55 years old)

‘I think occupational health hazards are not managed well and properly because sometimes you can see people or hear people reporting hazards later or even after a day or after two days being exposed.’ (P7, female, 59 years old)

The study findings highlight significant concerns regarding the inadequate management of occupational health hazards in healthcare settings, with participants expressing widespread dissatisfaction with the current state of hazard management.

#### Sub-theme 1.2: Inadequate training and education related to the management of occupational health hazards

The study examined the perceived factors related to training and education, which are crucial for managing OHH. As a result, one of the participants expressed the following concern about the lack of sufficient training and its impact on hazard management:

‘There is no in-service training conducted related to the management of occupational health services and there is no focal person.’ (P4, female, 55 years old)

‘To, me, education and training part on the management of occupational health hazards is done haphazardly. There is no consistency in the training and education of the employees in the ward, particularly in medical wards where nurses are at risk of being exposed to occupational health hazards. No regular training, training is only held as remedial once the incident has happened.’ (P7, female, 59 years old)

‘We are facing challenges regarding management regarding and prevention of hazards. There is no education and training about the management of occupational health hazard.’ (P8, male, 54 years old)

The study reveals significant concerns regarding the lack of a consistent and structured training and education on the management of OHH. Participants highlighted that in-service training is either non-existent or poorly coordinated, leading to a reactive approach to hazard management rather than proactive prevention.

#### Sub-theme 1.3: Lack of awareness on the management of occupational health hazards

Participants in this study expressed a lack of awareness regarding the management of OHH. This is evidenced by the following quotes:

‘There is no awareness is being raised, and however, nurses are only receiving limited information about occupational health services regarding management of occupational health hazards.’ (P1, female, 49 years)

‘The perception I have is that staff members are not aware of the risks in the department regarding occupational health hazards, specifically in terms of safety for nurses and patients; there is a lack of awareness being created.’ (P2, female, 46 years)

The study highlighted that nurses receive limited information on OHH, and awareness is not being effectively raised within the health facility.

#### Sub-theme 1.4: Absence of policies and guidelines related to the management of occupational health hazards

Participants have lamented the absence of policies and guidelines related to the management of OHH. This was evidenced by the following quotes:

‘So far in this hospital, only general in-services training related to infection control has been conducted but the training is not focusing on the management of occupational health hazard.’ (P1, female, 49 years old)

‘There is no occupational health hazards policy and guideline available, that I am familiar but all I know is there is division in the Labor Act which is talking about the welfare of the nurses.’ (P8, female, 54 years old)

Participants emphasised that the lack of clear, structured policies and protocols hampers effective hazard management in healthcare settings.

### Theme 2: Experiences occupational health hazards in health facilities

Most of the participants alluded to having experienced the following OHH in health facilities while working in the hospital: biological, psychological and physical hazards.

#### Sub-theme 2.1: Biological hazards

Several participants have conveyed their dissatisfaction by emphasising their experiences and concerns regarding biological hazards in the workplace:

‘Types of occupational health hazards that are experienced in this hospital are needle prick injuries and blood splash injuries which negatively affect the nurses, particularly ART initiation side effects.’ (P1, female, 49 years old)

‘In our ward, we are experiencing needle stick injuries because we are working with patients particularly when we are giving injections, we are experiencing splashing of blood exposure to body fluids such as ascites which exposure us to communicable diseases such as TB and meningitis. We have a lot of occupational health hazards in the medical ward and splashing of blood is putting nurses at risk of contracting Hepatitis B and HIV infection.’ (P4, female, 55 years old)

‘I have experienced needle pricks, but nurses are reporting later. Sometimes needle pricks are reported when a nurse sees a patient’s HIV status, particularly when a patient is HIV positive, and for falling, they only report when they experience pain. I am also experiencing Amniotic fluid splash during delivery.’ (P7, female, 59 years old)

Participants expressed significant concerns about the biological hazards they face in healthcare settings, particularly needle prick injuries and blood splash exposures while they are working with patients. This exposes nurses to communicable diseases.

#### Sub-theme 2.2: Psychological hazards

Regarding psychological hazards, participants expressed various concerns about various stressors in the workplace:

‘Stress in this hospital is extremely high, nurses are stressed and devastated. The environment is not conducive, and they cannot breathe.’ (P6, female, 55 years old)

‘Usually, the affected person would be worried that she or he might contract the disease. In the second place, the person would leave the workplace to look for treatment. In the process, the affected person usually gets prophylaxis for treatment, and this prophylaxis is associated with side effects as a result the nurses will be booked off from work.’ (P7, female, 59 years old)

‘The injured nurse usually goes through counselling in case of needle pricks, on the other hand, nothing is done to the substitution nurses and other occupational injuries nothing being done to support them.’ (P8, male, 54 years old)

Participants reported high-stress levels and a challenging work environment, with a lack of support for nurses experiencing occupational injuries. Counselling was offered only for needle pricks, and no psychological support was provided to substitute nurses or those with other OHH.

#### Sub-theme 2.3: Physical hazards

Participants perceived physical OHH with the following complaints:

‘The most common occupational health hazards that I observed and that have I have been reported in the past 6 months were, slippery because of wet floors, failure to observe warning signs injury is needle pricks.’ (P2, female, 46 years old)

‘Here in the hospital, nurses in medical wards are at risk of being beaten by mentally ill patients, because our hospital does not have seclusion rooms. If such types of patients are admitted, they are just running around and very aggressive because there are no seclusion rooms to contain them while they are waiting to be transferred to Oshakati Ward 16 psychiatric. So, nurses are, are exposed to physical hazards.’ (P6, female, 55 years old)

‘We are experiencing falling as part of a hazard because the floor is slippery. Consequently, this happens when the floor is wet, and the housekeeper fails to put up a warning sign when the floor is wet.’ (P7, female, 59 years old)

Participants reported various physical OHH, including slippery floors because of wet conditions and the failure to observe warning signs and the risk of harm from mentally ill patients, as the hospital lacked isolation rooms, leaving nurses exposed to potential physical harm.

### Theme 3: Challenges faced by nurses in managing occupational health hazards in health facilities

Participants indicated several challenges faced by nurses in managing OHH in healthcare facilities effectively. During data analysis, a subtheme was identified that captures these challenges.

#### Sub-theme 3.1: High prevalence of occupational health hazards

Regarding the prevalence of OHH, some participants expressed concerns about the following issues:

‘The management of occupational health hazards in the organization is crucial. However, the prevalence of occupational health hazards among nurses in hospitals is notably high.’ (P1, female, 49 years old)

‘I believe that our hospital needs to improve on the management of occupational health because the management of occupational health services is poor in this hospital and the prevalence of occupational health hazards is very high.’ (P2, female, 46 years old)

Moreover, another participant agreed with the previous statements, expressing similar concerns. By reporting the following sentiments:

‘In my view, the management of occupational health hazards among nurses is insufficient, and the prevalence of these hazards remains notably high.’ (P6, female, 55 years old)

‘In my personal view, occupational health hazards are poorly managed, as reports of hazards are often delayed, sometimes occurring one or two days after the incident, contributing to their high prevalence.’ (P7, female, 59 years)

#### Sub-theme 3.2: Shortage of human and material resources

Participants reported significant deficiencies in human and material resources, particularly highlighting shortages in personnel and essential equipment, as demonstrated by the following quotes:

‘We need support with gloves and syringes. We don’t have enough material resources; we are currently struggling with gloves and other supplies. There’s also a lack of aprons and gowns. Sometimes, when we place an order, we only receive one pack of aprons or nothing is given.’ (P4, female, 55 years old)

‘Our ward is facing a staff shortage, with a high patient-to-nurse ratio:50 patients: 7 staff, leading to stress and potential depression among staff. The hospital needs to increase its budget to hire more staff, purchase necessary materials, and recruit a person to manage occupational health hazards. Additionally, there is a lack of space and proper ventilation, and nurses have no designated rest area, forcing them to share a crowded tearoom with medical and nursing students.’ (P6, female, 55 years old)

‘Nurses need uniforms as part of their Personal Protective Equipment [*PPE*], which is required by law. The Labor Act mandates that employers provide PPE, including uniforms, at no cost. However, nurses at this hospital have been required to purchase their uniforms despite this legal obligation.’ (P8, male, 55 years old)

When participants were asked to describe the effects of material shortages in their departments, several nurse managers provided the following responses:

‘The shortage of necessary materials is a significant issue. We are exhausted from having to go back and forth to the neighbour ward to ask for gloves, face masks, and aprons to carry out tasks like dressing, bedmaking, and full patient washing.’ (P5, female, 58 years old)

‘Hospital management should hire wellness officers to oversee occupational health injuries and nurses’ well-being and increase nursing staff to better manage health hazards.’ (P6, female, 55 years old)

The study findings indicated a critical shortage of both human and material resources within the hospital. These deficiencies contribute to significant stress and potential burnout among nursing staff.

#### Sub-theme 3.3: Non-adherence to safety measures among nurses

Participants expressed their concern regarding the non-adherence to safety measures among nurses, as evidenced by the following quotes:

‘Although nurses are provided with personal protective equipment [*PPE*] and are encouraged to use it effectively, non-compliance with established policies, guidelines, and precautions has resulted in a high incidence of occupational health hazards.’ (P1, female, 49 years)

‘Nurses should be actively encouraged to report injuries; however, some may hesitate due to concerns about potential criticism from supervisors and the side effects associated with post-exposure prophylaxis [*PPE*]. This reluctance to report injuries can result in underreporting, thereby posing risks to both patient safety and the well-being of healthcare workers.’ (P3, female, 49 years old)

One participant also harmonised with this finding, as evidenced by the following:

‘Nurses must adopt a more positive attitude towards managing occupational health hazards and ensure compliance with policies and guidelines related to their management.’ (P2, female, 46 years old)

#### Sub-theme 3.4: Overcrowding of student nurse

Participants reported that overcrowding in clinical areas negatively impacted their clinical learning opportunities. As a result, several participants expressed the following concerns about this issue:

‘Due to the hospital’s role as a training facility, I have observe overcrowding in the theatre with students. I have observed several student nurses not wearing face masks outside the operating room. This behaviour contributes to the spread of airborne infections among students, which could potentially infect patients during surgery.’ (P2, female, 46 years old)

‘This hospital encounters difficulties in effectively supporting the large number of students assigned to it. Therefore, I respectfully urge all training institutions to implement a limit on the number of students allocated to this hospital.’ (P3, female, 49 years old)

This result aligns with another participant’s perspective, who mentioned that:

‘This hospital, serving as a training facility, faces overcrowding of students, resulting in inadequate supervision and compromised patient care. The high number of student population poses occupational health risks to nurses, while poor ventilation increases infection risks. Consequently, students acquire limited technical skills and graduate with insufficient practical experience, undermining the quality of nursing education, nurses’ health, and patient care.’ (P6, female, 55 years old)

Overcrowding of students in clinical areas negatively affects both clinical learning and patient care. This leads to inadequate supervision and increased health risks for nurses. Poor ventilation and lack of proper precautions further contribute to occupational health risks. To ensure a safer environment and more effective learning environment, training institutions should consider limiting the number of students allocated to this hospital.

## Discussion

This study explored and described nurse managers’ perceptions of managing OHH at the Intermediate Hospital Onandjokwe. Participants identified several key issues, which were categorised into themes: inadequate management of OHH, insufficient training and education on OHH management, lack of awareness regarding OHH and the absence of policies and guidelines. The sub-themes related to these concerns are discussed in detail in the following paragraphs.

These study findings concurred with the ILO (2019) when demonstrating the inadequate management of OHH when an organisation failed to identify, assess, control and monitor potential health risks in the workplace effectively. This can result in increased incidents of work-related injuries, illnesses and long-term health issues for employees. Another distinction, Lopez-Gómez et al. (2021) advocated that OHH at the workplace can be managed if best practices are implemented at the workplace, namely workplace integrated safety and health (WISH) and leading health metrics (LHM). The effects of OHH among nurses are a high rate of mortality, morbidity and premature age (Selvi 2020). The WHO and the ILO have established guidelines for implementation in health facilities to mitigate hazards among nurses, as the proper management of OHH is crucial for promoting an efficient and productive healthcare workforce. These standard guidelines include strategies such as elimination, substitution, engineering controls, administrative controls and PPE (International Labour Organization 2019). It has been proved that adequate management of OHH can be achieved if all nurses are protected from accidents and diseases that may arise from the institution. Hence, the evidence presented has shown that hospital management must comply with policies, protocols and guidelines related to the management of OHH. Awareness and training on occupational disease and injury can be used to familiarise nurses with information about the management of OHH. This training must cover the following topics: emergency and first aid procedures, occupational health and safety responsibility and reports on injuries and disease (International Labour Organization 2016).

Education and training are imperative to manage occupational hazards by equipping nurses to work safer and be more productive. Equally important to training and education of the nursing staff is equipping nurses with the skills of investigation of accidents, stress management and health promotion (Shaheen et al. 2023).

Furthermore, Alhassan and Poku (2018) advocated for monthly training and education sessions to support nurses and avoid OHH. In the final analysis, Rayan, Adam and Abdrabou (2021) emphasised the importance of education and training in OHH management to ensure and enhance work risk management. This includes improving managers’ and nurses’ performance. Managers must be familiar with regulations and implement efficient occupational health management practices. Nurses also need to be knowledgeable about safe work procedures and reporting for occupational hazards. Burton (2010) supports the idea that a healthy workplace model can enhance nurses’ health and safety. This model takes into consideration the following avenues of influence for a healthy workplace: physical work environment and psychological work environment, health resources in the workplace and enterprise community involvement.

Therefore, awareness of occupational hazards is crucial for nurses’ lives and for ensuring quality patient care. Additionally, awareness empowers nurses to implement best practices in the hospital for safe work. This can be achieved by providing nurses with skills in preventative measures for injuries and illnesses associated with work, along with creating suitable workplace conditions to optimise nurse health, which can raise awareness about managing OHH (Lopez-Gómez et al. 2021). Despite this factor, lack of awareness and absence of on-the-job training can contribute to the hazard. In addition, failure to focus on risk assessment, inferior management and lack of infection control will negatively contribute to maximising potential hazard exposure (Rayan et al. 2021).

The National OSH policy aims to prevent and manage workplace risks and hazards to protect and improve the social, physical and mental well-being of all workers, including nurses (MLIREC 2021). The policy also considers the ILO standards for occupational safety and health, highlighting international codes of practice for occupational health and safety programmes for nurses at the national, regional and facility levels. It offers legal frameworks and core principles, compliance with international standards, and ensures workers’ rights in the workplace (International Labour Organization 2019).

The implementation of policy and standards enables workers to perform their duties in a safer and secure environment and free from any hazards (World Health Organization 2021). On the one hand, work-related injuries can negatively affect a nurse’s quality of life and force them into early disability leave. Consequently, it can lead to psychological issues; a decline in business productivity; jeopardise the health and quality of care provided to patients by nurses, resulting in financial loss and damage an organization’s reputation, all of which are difficult to repair (International Labour Organization 2016). To end, the International Organization of Employers (2023) emphasised that the effective implementation of policy and guidelines in the management of OHH can guarantee the highest level of the workplace. The WHO and ILO have stipulated guidelines to be implemented in health facilities to mitigate hazards among nurses as proper management of OHH in healthcare facilities is very imperative to promote an efficient and productive healthcare force. Moreover, the following standard guidelines can be used as elimination, substitution, engineering control, administrative control and PPE (World Health Organization and International Labour Organization 2022). Adequate management of OHH can be achieved if all nurses are protected from accidents and diseases that may arise from the institution. The evidence presented has shown that hospital management must conform to OHH management policies, protocols and guidelines. It is evident that awareness and training on occupational disease and injury can be used to acquaint nurses with knowledge of the management of OHH, and this training must include the following aspects: emergency and first aid proceedings, occupational health and safety responsibility and reports on injuries and diseases acquired (Franklin & Gkiouleka 2021).

Psychological hazards in the nursing profession can have significant negative effects on nurses’ well-being and their ability to provide quality care (Ndejjo et al. 2015). The psychological hazards negative impact on the organisation can decrease productivity and absenteeism, affect the quality of work, cause a high turnover of staff and increase the risk of injury (Griffin 2022). Amare et al. (2021) concluded that physical hazards can cause both short-term and long-term effects, varying from mild to severe, including death, psychological distress and temporary or permanent impairments. These hazards can also lead to negative effects, low morale and reduced productivity because of trauma. Psychological trauma, alongside factors such as absenteeism, family conflict, job stress, low management trust and nurse turnover, can have significant consequences in the workplace. Despite the implementation of risk management strategies, it remains essential to maintain continuous communication with nurses regarding potential risks. This should be achieved through a proactive approach that consistently evaluates the likelihood and severity of workplace injuries, illnesses and property damage (Gan 2019).

The high prevalence of OHH can result in various consequences, such as trauma, post-traumatic stress disorder (PTSD), anxiety, depression, loss of dignity, decreased self-esteem, suicide attempts, premature ageing, loss of autonomy, injuries, absenteeism and a lack of trust in others (Aldhaen 2022). A shortage of human and material resources can lead to delays, decreased productivity and overall inefficiency (Odigwe & Owan 2022). Non-compliance with safety regulations can result in serious consequences, such as fines, legal action and damage to a company’s reputation. More importantly, it can lead to accidents and injuries, which can result in physical harm, lost productivity and increased costs for medical treatment and workers’ compensation (Nwankwo, Karanja & Vasanthakaalam 2017). Compliance with safe measures related to the management of OHH and safety measures can result in the reduction of workplace injuries and fatalities (Afework, Tamene & Tafa 2024). As a result, employers and employees must adhere to health and safety measures, such as wearing protective gear during work, to reduce economic losses. Additionally, organisations are required to implement and enforce occupational health and safety policies, guidelines and programmes to minimise work-related injuries and diseases (Griffin 2022).

Savioli et al. (2022) agree with previous studies, highlighting the negative effects of student congestion, such as overcrowding, limited resources and insufficient individualised attention. These factors harm the psychological well-being and quality of life of both nurses and students, ultimately hindering the learning process. Savioli et al. (2022) agreed with the study’s findings by emphasising the negative impacts of student congestion, including overcrowding, limited resources and inadequate individualised attention. These factors harm the psychological well-being and quality of life of nurses. They recommend preventing overcrowding in clinical settings to create a supportive clinical environment. Additionally, Berhe and Gebretensaye (2021) emphasise that a positive working environment is cultivated through adequate clinical supervision, constructive feedback and impartial assessments by nurses. Furthermore, Savioli et al. (2022) underscore the importance of optimal student allocation, noting that maintaining an appropriate student-to-nurse ratio enhances both the physical and psychological health of nurses.

### Limitations of the study

The study focused on nurses in managerial positions at the Intermediate Hospital Onandjokwe using purposive sampling, limiting its findings to a specific context and making them non-generalisable to all nurse managers in Namibia. Additionally, as the research was conducted in a single public hospital, its broader applicability is restricted. Despite these limitations, the study offers valuable insights for scholarly and future research.

### Recommendations

Employers must allocate adequate resources for effective OHH management, including PPE, infrastructure and human resources while enforcing clear policies and standardised practices for hazard management. Comprehensive training should be provided to educate employees on recognising and reporting hazards, and a supportive environment should encourage accurate hazard reporting and constructive feedback. Nurses are expected to follow safety protocols, engage in training and report hazards promptly to prevent future issues.

## Conclusion

The nurse managers at the Intermediate Hospital Onandjokwe perceived the management of OHH extremely poor. They experience a diversity of hazards, including physical, chemical, biological and psychosocial risks. Contributing factors to the hazards were insufficient resources, the absence of an occupational health services policy and a lack of training. These deficiencies result in a poorly managed work environment where hazards are not effectively addressed. Furthermore, the study revealed a concerning trend of under-reporting OHH, with nurses hesitant to report incidents because of fears of stigmatisation and professional consequences. This issue was particularly evident when a patient’s human immunodeficiency virus (HIV) status became known later, leading to delayed reporting and complicating hazard management. Such delays are critical as nurses require post-exposure prophylaxis (PEP) treatment to prevent HIV infection through blood exposure.
